# mDixon ECG-gated 3-dimensional cardiovascular magnetic resonance angiography in patients with congenital cardiovascular disease

**DOI:** 10.1186/s12968-019-0554-3

**Published:** 2019-08-08

**Authors:** Soultana Kourtidou, Marty R. Jones, Ryan A. Moore, Justin T. Tretter, Nicholas J. Ollberding, Eric J. Crotty, Mantosh S. Rattan, Robert J. Fleck, Michael D. Taylor

**Affiliations:** 1 Weil Cornell Medicine, Department of Pediatrics, Pediatric Cardiology, 525 East 68th St, F-677, New York, NY 10065 USA; 2 The Heart Institute, Department of Pediatrics, David’s Medical Center, 919 East 32nd Street, Austin, TX 78705 USA; 3 Department of Radiology, Department of Pediatrics, Cincinnati Children’s Hospital Medical Center, 3333 Burnet Ave, Cincinnati, OH 45229 USA; 4 Division of Biostatistics and Epidemiology, Department of Pediatrics, Cincinnati Children’s Hospital Medical Center, 3333 Burnet Ave, Cincinnati, OH 45229 USA; 5 St. David’s Medical Center, 919 East 32nd Street, Austin, TX 78705 USA

**Keywords:** Cardiovascular Magnetic Resonance (CMR), Contrast Enhanced Magnetic Resonance Angiography (CE-MRA), Congenital heart disease (CHD), Aortopathy, balanced Steady-State-Free Precession (bSSFP), modified Dixon (mDixon)

## Abstract

**Background:**

Cardiovascular magnetic resonance (CMR) angiography (CMRA) is an important non-invasive imaging tool for congenital heart disease (CHD) and aortopathy patients. The conventional 3D balanced steady-state free precession (bSSFP) sequence is often confounded by imaging artifacts. We sought to compare the respiratory navigated and electrocardiogram (ECG) gated modified Dixon (mDixon) CMRA sequence to conventional non-gated dynamic multi-phase contrast enhanced CMRA (CE-CMRA) and bSSFP across a variety of diagnoses.

**Methods:**

We included 24 patients with CHD or aortopathy with CMR performed between September 2017 to December 2017. Each patient had undergone CE-CMRA, followed by a bSSFP and mDixon angiogram. Patients with CMR-incompatible implants or contraindications to contrast were excluded. The studies were rated according to image quality at a scale from 1 (poor) to 4 (excellent) based on diagnostic adequacy, artifact burden, vascular border delineation, myocardium-blood pool contrast, and visualization of pulmonary and systemic veins and coronaries. Contrast-to-noise ratio (CNR), signal-to-noise ratio (SNR) and quantitative vascular measurements were compared between the two gated sequences. Bland-Altman plots were generated to compare paired measures.

**Results:**

All scans were diagnostically adequate. Mean (SD) quality scores were 3.4 (0.7) for the mDixon, 3.2 (0.5) for the bSSFP and 3.4 (0.5) for the CE-CMRA. Qualitatively, the intracardiac anatomy and myocardium-blood pool definition were better in the bSSFP; however, mDixon images showed enhanced vessel wall sharpness with less blurring surrounding the anatomical borders distally. Coronary origins were identified in all cases. Pulmonary veins were visualized in 92% of mDixon sequences, 75% of bSSFP and 96% of CE-CMRA. Similarly, neck veins were identified in 92, 83 and 96% respectively. Artifacts prevented vascular measurement in 6/192 (3%) and 4/192 (2%) of total vascular measurements for the mDixon and bSSFP, respectively. However, the size of signal void and field distortion were significantly worse in the latter, particularly for flow and metal induced artifacts.

**Conclusion:**

In patients with congenital heart disease, ECG gated mDixon angiography yields high fidelity vascular images including better delineation of head and neck vasculature and pulmonary veins and fewer artifacts than the comparable bSSFP sequence. It should be considered as the preferred strategy for successful CHD imaging in patients with valve stenosis, vascular stents, or metallic implants.

**Electronic supplementary material:**

The online version of this article (10.1186/s12968-019-0554-3) contains supplementary material, which is available to authorized users.

## Background

Cardiovascular magnetic resonance (CMR) angiography (CMRA) is a standard modality used in the routine evaluation of pediatric and adult patients with congenital heart disease (CHD) or aortopathy [1–5]. Conventional dynamic contrast enhanced (CE)-CMRA provides high-fidelity three-dimensional (3D) visualization of the heart and chest vasculature without exposure to ionizing radiation or invasive catheterization [6].

Dynamic CE-CMRA is a well-established extensively validated technique proven to be accurate in CHD [3–9]. However, the need for cardiac gated images promoted the development of contemporary electrocardiogram (ECG)-triggered and respiratory gated pulse sequences [10, 11]. The 3D balanced steady state free precession (bSSFP) CMRA, often termed “whole-heart” imaging, is an ECG-gated and respiratory-navigated sequence with high signal to noise ratio (SNR) for delineating both intracardiac and extracardiac anatomy [12]. Since the bSSFP contrast is a function of the T2/T1 ratio between tissues, fat appears bright and magnetization preparation schemes are necessary to achieve adequate fat suppression [12]. 3D bSSFP is currently the preferable sequence for coronary artery visualization in CHD [13–15]. Its main disadvantage is sensitivity to flow and susceptibility artifacts [16]. This is can be important to CHD patients who often have both valvar and vessel abnormalities or implanted ferromagnetic devices.

Unlike bSSFP, fat-water separation Dixon-based methods include the underlying B_0_ distribution in the signal model; therefore image acquisition is less susceptible to field inhomogeneity [16–19]. Modified Dixon (mDixon) CMRA has excellent image quality at 1.5 T and improve vessel-lumen to fat contrast [20], assist in identifying intramyocardial fatty infiltration in arrhythmogenic right ventricular cardiomyopathy [21, 22], quantify pericardial and epicardial fat in patients with high-risk cardiovascular disease profile [23], and help in the characterization of peripheral arterial occlusive disease [24, 25]. mDixon strategies to reduce artifacts introduced by metal-implants have been a primary focus of musculoskeletal MRI following hip-replacement surgery [26].

To date no studies have reported the application of mDixon CMRA to the evaluation of CHD patients who also often have metalic implants. We sought to investigate the utility of this respiratory navigated and ECG gated mDixon CMRA sequence across a variety of CHD diagnoses and aortopathies in a patient population of a wide range of ages and sizes. We hypothesized that the mDixon sequence would provide high quality diagnostic images with fewer artifacts compared to the conventional whole-heart bSSFP approach.

## Methods

We conducted a retrospective study comparing three different CMRA sequences in CHD patients presenting for CMR evaluation at our institution between September 2017 to December 2017. Consecutive patients with CHD or aortopathy, who had CMR studies with all three sequences were included irrespective of age (children and adults) or diagnosis. We excluded patients with non-CMR compatible implants or contraindications to contrast. The study was approved by our Institutional Review Board which waived informed consent.

### CMRA protocol

The CMR studies were performed in a clinical setting using a 1.5 T whole-body CMR scanner (Ingenia; Philips Healthcare, Best, The Netherlands) using a standard clinical protocol including dynamic CE-CMRA, Respiratory navigator and ECG gated bSSFP and mDixon whole-heart sequences. Safety screening, compatible monitoring equipment, appropriate imaging coil and cardiac anesthesia for infants requiring sedation were applied in accordance with guideline-directed standards. All studies were performed using contrast (Gadoterate meglumine) at a dose of 0.2 ml/kg administered by power injector or manually at a flow rate of 2–3 ml/sec depending on patient age and intravenous catheter size. Each patient underwent a non-gated dynamic multi-phase CE-CMRA followed by the bSSFP and the mDixon pulse sequences.

### Sequences

All three CMRA sequences have been previously described in detail [3, 5, 8–10, 15, 17–19, 27, 28]. Conventional, dynamic CE-CMRA was performed without ECG gating. Five dynamic phases were acquired beginning 8–12 s after contrast injection. The first 2–3 dynamic phases were acquired during an inspiratory breath hold. Dynamic times were 6–10 s in length. The bSSFP and mDixon sequences were acquired using a beam shaped navigator placed over the hemi-diaphragm opposite the heart. Both sequences were ECG-gated to mid-diastole or end-systole for younger patients with higher heart rates. The 3D-bSSFP is a standard balanced gradient echo steady state precession pulse sequence with a T2-preparation pre-pulse and a spectral pre-saturation pulse. The mDixon sequence is an unbalanced fast gradient echo sequence that achieves fat suppression through chemical shift-based water-fat separation with the modified Dixon technique [19]. The typical acquisition parameters for the whole-heart CMRA protocols are summarized on Table 1. Limited patient-specific adjustments were applied as needed to optimize image acquisition and scan time. The shot durations were decreased to 80 ms for systolic acquisitions. Three-dimensional volume datasets were obtained in the coronal plane covering the chest, the lower neck vasculature and the upper abdomen.

**Table 1:** Reference Acquisition Parameters for Contrast Enhanced Cardiovascular Magnetic Resonance (CE-MRA), 3D whole-heart Balanced State Free Precession (bSSFP) and modified Dixon (mDixon) sequences

Parameter	CE-CMRA	bSSFP	mDixon
Average Acquisition Time Goal	Dynamic scan time goal ~ 12 s	3:11 min	3:08 min
TE (ms)	0.87	2	1st image: 1.8 2nd image: 3.4
TR (ms)	2.2	4.5	5.2
Flip Angle (degrees)	30°	90°	15°
FOV (mm)	260 × 260	260 × 260	260 × 260
Acquired Voxel Size	1.4 × 1.4 × 2.8	1.4 × 1.4 × 1	1.4 × 1.4 × 1
Recon Voxel (mm^3^)	0.9 × 0.9 × 0.4	0.9 × 0.9 × 1	0.9 × 0.9 × 1
Sense	LR: 2.5; AP: 1.0	LR: 1.5; AP: 1.0	LR: 2.0; AP: 1.5
Trigger Delay	NA	Mid-Diastole	Mid-Diastole
Shot Duration (ms)	NA	130	130
Navigator Window (mm)	Cartesian k-space acquisition window	7	7
Slice number	90	135	135
Bandwidth (Hz/pixel units)	0.165	0.35	0.3

*Abbreviations*: *mDixon* modified-Dixon, *bSSFP* balanced Steady State Free Precession, *CE-CMRA* Contrast-Enhanced Cardiovascular Magnetic Resonance Angiography; TE echo time; TR repetition time; NA not applicable; LR Left Right; AP Anterior Posterior

### Image analysis

The primary image analysis was performed using the phase with the densest arterial contrast for the dynamic CE-CMRA and the water images of the mDixon acquisition. The initial analysis required comparing the mDixon in-phase images and water images for both qualitative and quantitative image quality. The water images were chosen for the primary analysis based their significantly higher SNR and contrast to noise ratio (CNR), better image quality, and improved vessel delineation secondary to effective and homogenous fat suppression (Additional file 1: Figure S1, Additional file 2: Figure S2, and Additional file 3: Figure S3). Two readers (MT; SK) reviewed and graded the studies independently according to overall quality (1-poor, 2-diagnostically adequate, 3-good, 4-excellent). The scoring parameters were: diagnostic adequacy, presence and effect of artifacts, quality of anatomical borders (blurred versus sharp), myocardium-blood pool contrast, ability to visualize the pulmonary veins, neck veins and coronaries. CNR and SNR were compared in the arterial blood pool and the myocardium, using the equivalent noise in the lungs for reference [20]. Considering the importance of obtaining precise vascular measurements in the CHD population, we performed cross-sectional vascular lumen measurements for the aortic root, ascending aorta, main pulmonary artery (MPA) (or the pulmonary conduit when indicated), left and right branch pulmonary arteries (LPA, RPA), aorta at the level of isthmus, right superior vena cava (R-SVC) and the left anterior descending coronary artery (LAD). Vascular measurements were performed on multiplanar reformatted images in accordance with the 2015 recommendations of the Society for Cardiovascular Magnetic Resonance (SCMR) for obtaining CMR values in adults and children [29]. The measurement location remained consistent among the three sequences independent of image quality. In patients evaluated for known vessel abnormalities, such as stenosis or dilation, the measurement was obtained from the respective area of interest. Quality scores for dynamic, non-gated CE-CMRA were assigned based on the best potential acquisition accounting for the limited intracardiac detail or coronary imaging. The total scan time was recorded for each sequence. Given the fundamental differences in acquisition time and contrast between breath-holding versus respiratory navigator gating techniques, CE-CMRA measurements were not included in the SNR, CNR, vascular and scanning time analyses.

### Statistical analysis

Categorical data were summarized using frequencies and percentages. Continuous values were expressed as mean and standard deviation (SD). Bland-Altman limits of agreement were used to assess the extent of agreement between bSSFP, mDixon and CE-CMRA methodologies. Means (SD), bias (95% CI), upper and lower limits of agreement for the vascular measures, image quality values and scanning time were calculated. Bland-Altman plots were generated to yield a visual comparison of the difference of paired measures against their averages. For binary outcomes, we examined the absolute number and proportion of observations agreeing. Raw-agreement and the weighted kappa statistic were computed to assess inter-rater agreement in the image quality scores. Finally, paired t-tests were applied to test differences in scan times between bSSFP and mDixon. Statistical significance was defined as a *P*-value of < 0.05. Analyses were conducted using R v3.4.0 and the tidyverse (v1.2.1), blandr (0.4.3), psych (v1.7.5), and cowplot (v0.9.2) packages.

## Results

Twenty-four consecutive qualifying CMR scans were included from September 2017 – December 2017. One patient was excluded due to disproportionately poor quality and inability to demonstrate any anatomic structures in the bSSFP sequence. Table 2 summarizes the baseline subject characteristics and CHD or aortopathy diagnoses. Ages ranged from 4 months to 40 years, with 50% of the patients ≤20 years. Fifteen out of the 24 participants were males. Three cases required cardiac anesthesia: a 4-month-old, a 4-year-old and a 19-year-old patient with Trisomy 21.

**Table 2:** Baseline Characteristics and Congenital Heart Disease diagnosis

Patient	Gender	Age	Diagnosis
#1	Female	4 mo	Scimitar syndrome, PAPVC, RPA branch supplying the left lower lung, ASD, VSD, dysplastic pulmonary valve
#2	Male	4 yrs	Heterotaxy, Right atrial isomerism, Complete AVSD, DORV, D-TGA, TAPVC, b/l SVC, s/ p TAPVC repair, b/l Glenn and pulmonary artery band
#3	Male	9 yrs	Loeys-Dietz Syndrome, s/p valve sparing aortic root replacement with coronary arteries re-implantation
#4	Female	13 yrs	TOF s/p VSD closure and transannular patch repair of the right ventricular outflow tract
#5	Female	13 yrs	TOF, AVSD, s/p patch repair, pulmonary valve regurgitation, b/l AVV regurgitation, ascending aorta dilation
#6	Female	14 yrs	TAPVC, Shone complex, hypoplastic MV, BAV, Coarctation of the aorta, s/p TAPVC repair, s/p coarctation repair with end to end anastomosis
#7	Female	14 yrs	TAPVC s/p repair with residual PAPVC
#8	Male	16 yrs	Hypoplastic Left Heart Syndrome, s/p Fontan procedure
#9	Female	16 yrs	DORV, subaortic VSD s/p patch VSD closure; s/p resection of subaortic membrane and RV muscle bundle
#10	Male	17 yrs	Congenital LPA hypoplasia
#11	Female	19 yrs	Pulmonary valve stenosis s/p balloon valvuloplasty
#12	Male	19 yrs	Down syndrome, Unrepaired Coarctation of aorta, congenital stenoses of the branch pulmonary arteries
#13	Male	20 yrs	TOF s/p VSD and transannular patch repair, mechanical pulmonary valve replacement, aortic root dilation
#14	Male	24 yrs	D-TGA s/p arterial switch; RPA narrowing, hypoplastic LPA, dilated aortic root, aortic valve regurgitation
#15	Male	24 yrs	TOF s/p complete repair, aortic root dilation, high LCA origin, pectus excavatum
#16	Male	27 yrs	Pulmonary vale atresia, VSD, discontinuous branch pulmonary arteries s/p repair
#17	Male	27 yrs	BAV, dilated aortic root
#18	Male	27 yrs	VSD, Coarctation of aorta s/p SCA flap repair, distal TAA pseudoaneurysm, dilated aortic root
#19	Female	27 yrs	Unrepaired PAPVC, BAV
#20	Male	30 yrs	BAV s/p repair with residual stenosis, ascending aorta dilation
#21	Male	31 yrs	BAV s/p bioprosthetic valve placement
#22	Male	38 yrs	TOF s/p RV-PA conduit replacement, with residual conduit stenosis and aortic root dilation
#23	Male	40 yrs	BAV, s/p mechanical valve placement, aortic root and ascending aorta dilation
#24	Female	40 yrs	Ehlers-Danlos syndrome s/p aortic root replacement; MRI-compatible Pacemaker

*Abbreviations*: *mo*: months, *yrs*: years, *PAPVC* Partial Anomalous Pulmonary Venous Connection, *RPA* Right Pulmonary Artery, *ASD* Atrial Septal Defect, *VSD* Ventricular Septal Defect, *AVSD* Atrio-Ventricular Septal Defect, *DORV* Double Outlet Right Ventricle, *D-TGA* D-Transposition of the Great Arteries, *TAPVC* Total Anomalous Pulmonary Venous Connection, *B/L* Bilateral, *SVC* Superior Vena Cava, *S/P* Status Post, *TOF* Tetralogy of Fallot, *AVV* Atrioventricular Valve, *MV* Mitral Valve, *BAV* Bicuspid Aortic Valve, *LPA* Left Pulmonary Artery, *SCA* Subclavian Artery, *TAA* Transverse Aortic Arch, *RV-PA* Right Ventricle to Pulmonary Artery

### Image quality

**Fig. 1:**
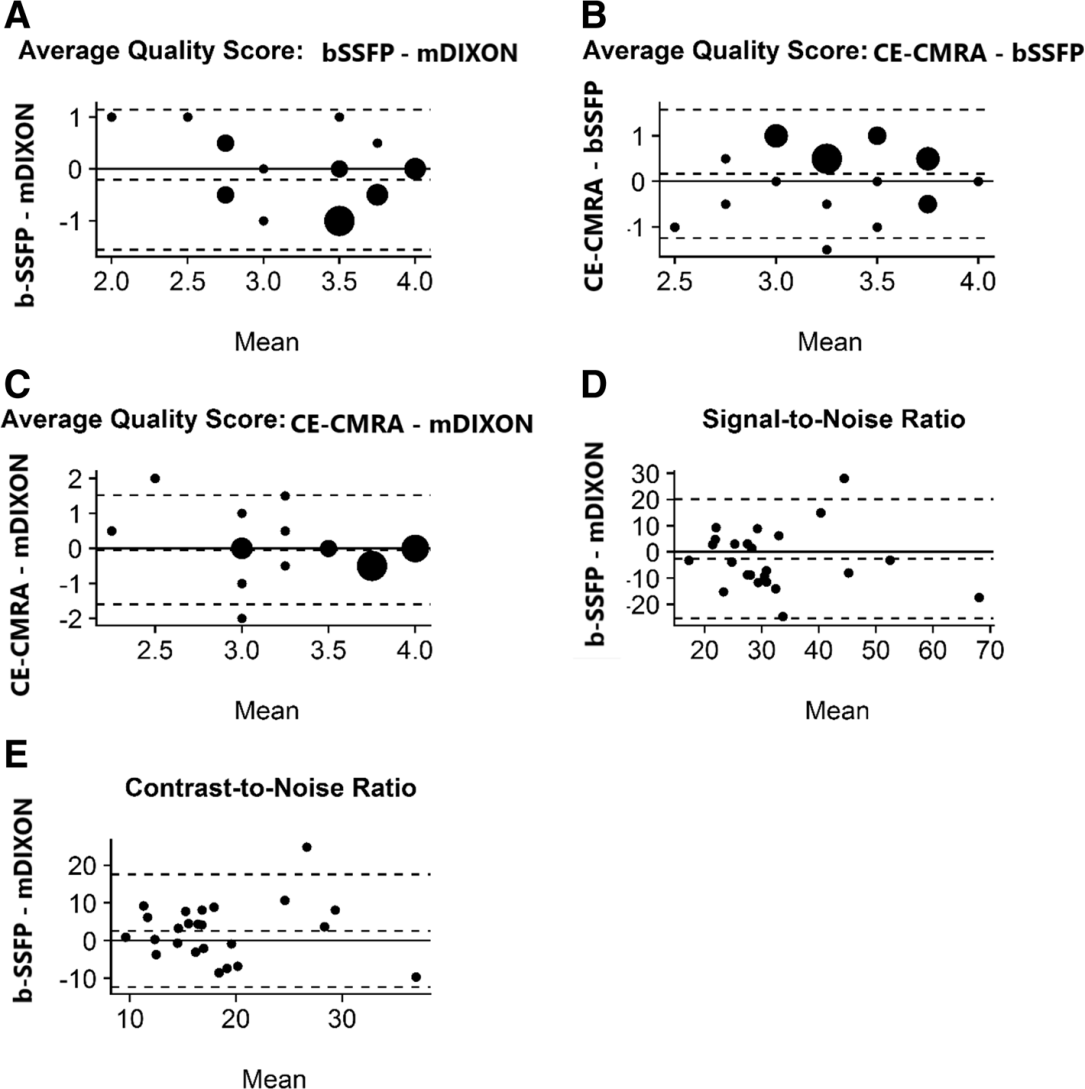
Bland-Altman Plots for Image Quality Scores between Balanced Steady State Free Precession (bSSFP)-modified Dixon (mDixon) (**a**), Contrast Enhanced (CE)-Cardiovascular Magnetic Resonance Angiography (CMRA)-bSSFP (**b**), CE-CMRA-mDixon (**c**); Signal-to-Noise Ratio (SNR) (**d**) and Contrast-to-Noise Ratio (CNR) (**e**) between bSSFP-mDixon. The y-axis provides the difference of the paired measures. The x-axis provides the mean of the paired measures. The dotted lines provide the mean difference and the 95% limits of agreement

The overall image quality of the mDixon water images was significantly better than the in-phase images. Consequently, the remainder of the results refers to the mDixon water images. The image quality of the mDixon images was comparable, and for certain features, superior to that of the bSSFP. All scans produced clinically diagnostic images with the majority of them rated as “good” or “excellent”. No water fat swaps were visible in the mDixon images. There were no anatomic discrepancies with the reported study findings. The Bland-Altman plots revealed acceptable agreement between the bSSFP-mDixon, CE-CMRA-bSSFP and CE-CMRA-mDixon pairs with respect to their average image quality scores (Fig. 1, a-c). The mean quality values, averaged for the two reviewers, were 3.4 (0.7) for the mDixon, 3.2 (0.5) for the bSSFP and 3.4 (0.5) for the CE-CMRA. The bias (mean of the observed differences) between mDixon and bSSFP was − 0.21(95% CI: − 0.50; 0.08) and the limits of agreement (LOA) and 95% CIs were − 1.56 (− 2.07; − 1.06) and 1.14 (95% CI: 0.64; 1.65) for the lower and upper LOAs, respectively. The bias (mean of the observed differences) between mDixon and CE-CMRA was 0.04 (95% CI: − 0.29; 0.38) and the LOA (95% CI) were − 1.51 (− 2.09; − 0.93) and 1.6 (1.02; 2.18) for the lower and upper LOAs, respectively. The weighted kappa statistic demonstrated variable inter-observer agreement between the two reviewers (MT, SK) depending on the sequence type. Specifically, it was good for the mDixon [raw:71%; weighted kappa = 0.62 (0.41, 0.83)], moderate for the bSSFP [raw: 63%; weighted kappa = 0.46 (0.22, 0.70)] and fair for the CE-CMRA [raw: 46%; weighted kappa = 0.37 (0.03, 0.70)].

**Fig. 2:**
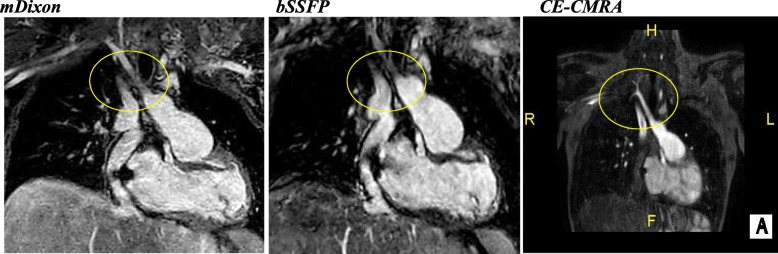
16-year-old with hypoplastic left heart syndrome s/p Fontan palliation. Demonstration of complex CHD anatomy by whole heart sequences and standard CE-CMRA. Smaller flow artifact in the Fontan fenestration, lung and neck vasculature, comparable quality of myocardium-blood boarder, sharper diaphragm/liver/heart, chest cavity boarders. Better SVC Fontan anastomosis seen in balanced SSFP. Less detailed visualization of the anatomy in the CE-CMRA

**Fig. 3:**
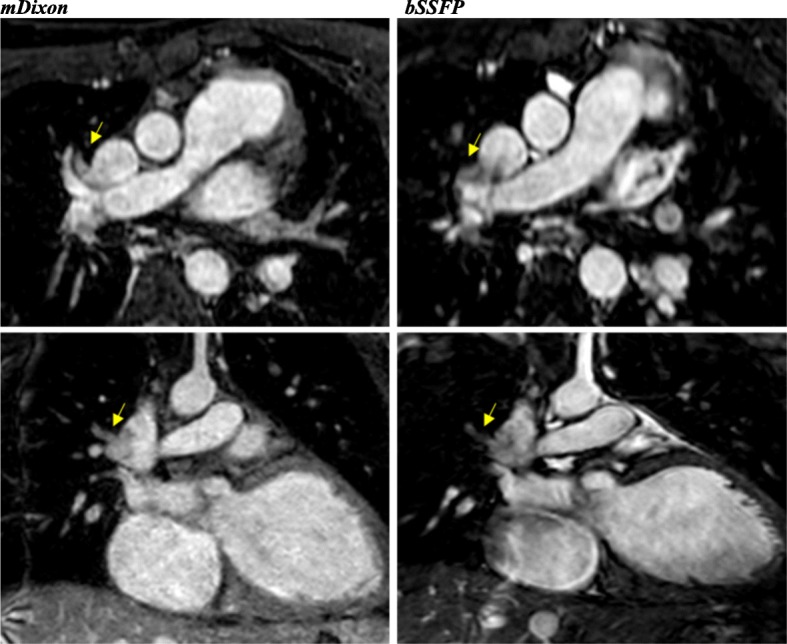
15-year-old girl with history of total anomalous pulmonary venous connection (TAPVC) s/p repair with residual partial anomalous pulmonary venous connection (PAPVC); the right upper pulmonary vein drains into the SVC. The abnormal course and connection (arrow) is only partially visualized in the bSSFP

**Fig. 4:**
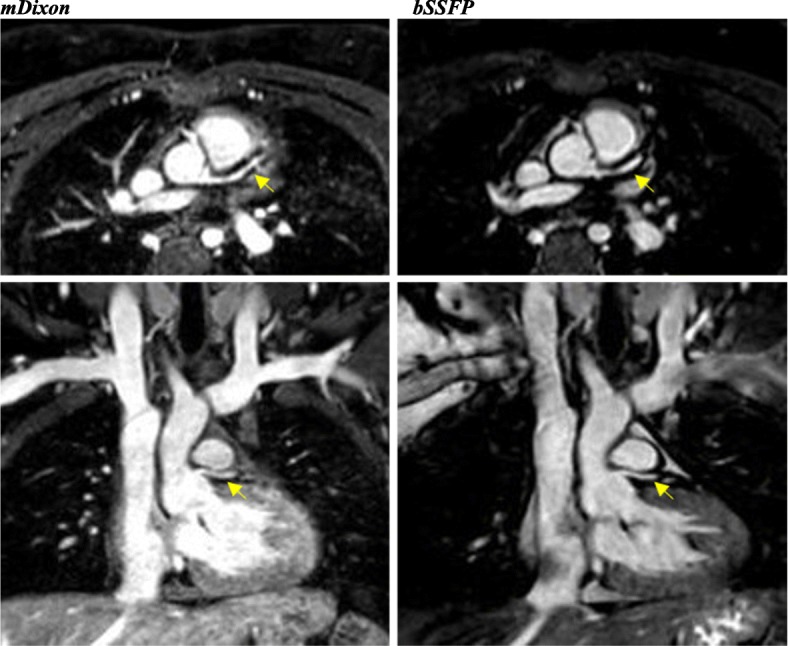
19-year-old female with unrepaired coarctation of the aorta. Comparable performance of the whole-heart sequences in demonstrating the origins and proximal coronary artery courses. Also, note the improved visualization of the neck and lung vasculature as well as the right hemidiaphragm in the mDixon image

**Fig. 5:**
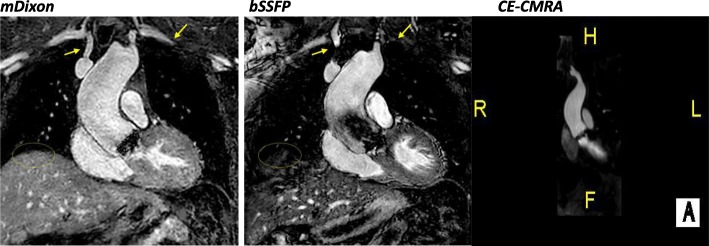
40-year-old male with history of BAV s/p mechanical valve (St. Jude) placement with aortic root and ascending aorta dilation. The flow artifact is significantly reduced in the mDixon image. The yellow arrows denote veins obscured by artifacts in the balanced SSFP image. Also, note the improved visualization of the right hemi-diaphragm in the mDixon image (Elliptical ROI). CE-CMRA phase focused in the aortic root and ascending aorta; small flow artifact

**Fig. 6:**
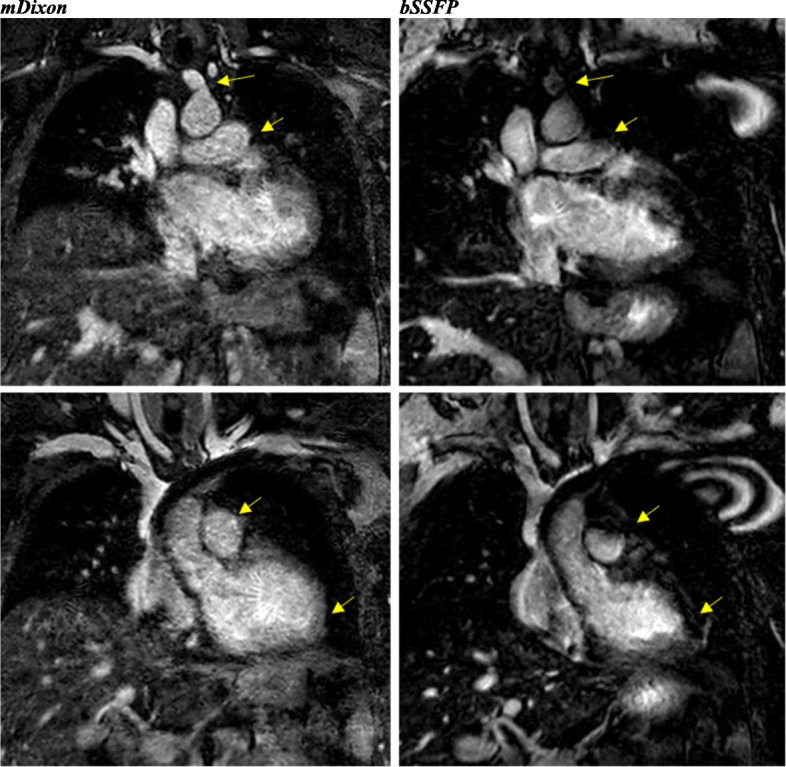
40-year-old with Ehlers-Danlos syndrome and left ventricular non-compaction, s/p CMR-compatible pacemaker. The image quality in both is degraded. The main pulmonary artery (MPA) and head and neck vasculature are better visualized in the mDixon image. However, the endomyocardial border is better defined in the bSSFP image, a common finding in the study

With regards to individual image quality elements, mDixon depicted the entire field of view better as compared to CE-CMRA and bSSFP, offering a comprehensive assessment of the neck vasculature, the chest cavity and the upper abdomen anatomy. Qualitatively, the intracardiac anatomy and myocardium-blood pool definition were better in the bSSFP; however, mDixon images showed superior vessel wall sharpness with less blurring in distal vessels, particularly in vessels near the edges of the field of view and vessels within the lung fields, *(*Fig. 2*).* In agreement with the above, pulmonary veins were visualized in 92% of the mDixon sequences, while the respective percentages were 75% for bSSFP and 96% for CE-CMRA, *(*Fig. 3*).* Adequate visualization of the neck veins was achieved in 92% of mDixon, 83% of bSSFP and 96% of CE-CMRA images, (Figs. 2, 3, 4, 5 and 6)*.* Notably, we were able to identify arterio-venous collateral vessels within the lungs, as well as, aortopulmonary collateral vessels in both mDixon and bSSFP images; yet, their distal courses were better displayed in the mDixon images. Both whole-heart sequences performed very well in demonstrating the coronary artery origin and distal courses, *(*Fig. 4*).* Thus, contrast intensity and incomplete fat suppression artifact subjectively interfered with quality and in 3 cases it did not allow visualization of LAD at its full length. Despite our observations, the Bland-Altman plots did not display any obvious differences for the SNR and CNR magnitude between bSSFP and mDixon, (Fig. 1, d-e). In Figs. 2, 4, and 5, the diaphragm is significantly easier to see in the mDixon images. This is likely secondary to B0 inhomogenities as the respiratory navigator respiratory gating was identical for both sequences.

### Artifacts

Of the 24 cases, 17 had some type of artifact in one or more sequences. Given the anatomic complexity and the high likelihood of prior surgical or catheter induced intervention, susceptibility and flow-related artifacts were the most frequently encountered types. Such artifact burden was notably less appreciable in mDixon versus bSSFP and CE-CMRA. The predominant artifacts in the bSSFP images were B0 inhomogeneities and flow artifacts; in the mDixon images they were B0 inhomogeneities and incomplete fat/water separation. Figures 5 and 6 show the impact of an artificial aortic valve and an CMR-compatible pacemaker, respectively, among the three corresponding techniques. Motion blurring was typically associated with the CE-CMRA images. mDixon demonstrated successful fat-suppression resulting to minimal chemical-shift artifact around the pericardial and vessel wall borders, including the coronary arteries. The presence of artifact prevented us from performing designated vascular measurements in 6/192 (3%) of cases in the mDixon and 4/192 (2%) in the bSSFP images, however, the size of the obscured area was significantly worse in the latter sequence. For the mDixon, of the 6 inadequately visualized vascular specific locations, two were obscured by metal artifact, one case was related to flow-artifact in the main pulmonary artery and 3 cases were attributed to limited visualization of coronary arteries secondary to the high contrast intensity or incomplete fat suppression. In contrast, susceptibility was the only type of artifact that limited our ability to perform vascular measurements in bSSFP.

A summary table with detailed Bland-Altman agreement estimates for all the assessed image quality parameters (Image quality score, SNR, CNR, artifact preventing the performance of vascular measurement, visualization of neck and pulmonary veins) is included in supplemental analysis (Additional file 4: Table S1 and Additional file 5: Table S2).

### Temporal order

**Fig. 7:**
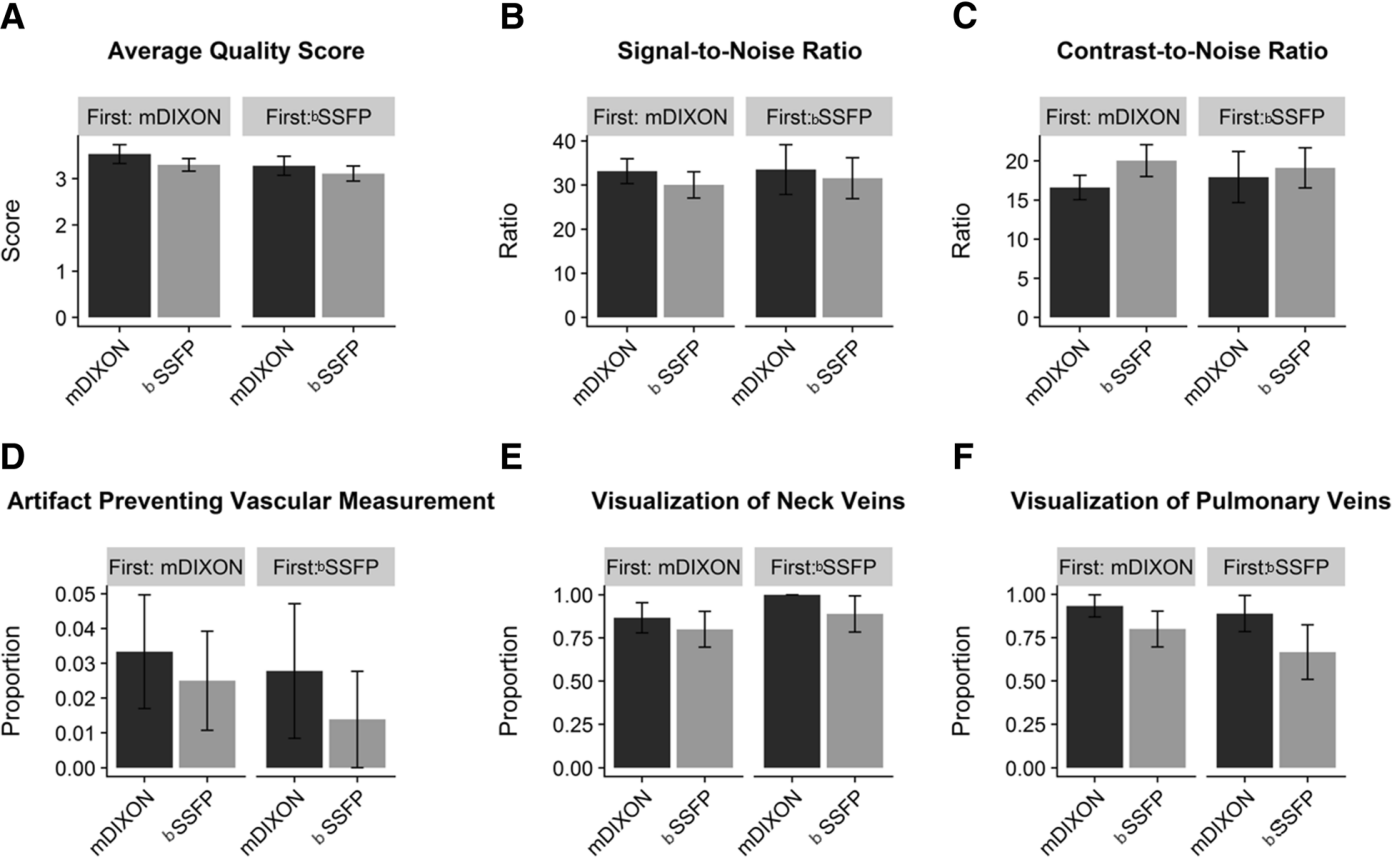
Effect of temporal order of sequence in image quality parameters: (**a**) Average quality score, (**b**) Signal-to-Noise Ratio, (**c**) Contrast-to-Noise Ratio, (**d**) Artifact Preventing Vascular Measurement, (**e**) Visualization of Neck Veins, (**f**) Visualization of Pulmonary Veins

Both whole-heart sequences were performed after contrast administration for the CE-CMRA. The mDixon preceded the bSSFP in 15 out of the 24 studies. No impact of the temporal order was detected for the image quality scores, SNR, CNR, presence of artifact preventing a measurement, or visualization of venous structures, (Fig. 7).

### Vascular measurements

We performed a comprehensive assessment of the chest vasculature to include both arterial and venous structures with wide-ranging sizes. Bland-Altman analyses for 8 cross-sectional vascular measurements are summarized in Table 3. The bias and 95% limits of agreement between each set of vascular diameters showed no systematic bias and less than 10% measurement difference between all mDixon and bSSFP estimates irrespective of the vessel type and size. The analysis was also performed using the mDixon water images. There was no systematic bias in the water and in-phase mDixon image measurements (Additional file 6: Table S3).

**Table 3:** Summary of agreement estimates for quantitative vascular measurements

Cross-sectional Vessel Diameter	mDixon Mean (SD)	bSSFP Mean (SD)	Bias (95% CI)	Lower LOA (95% CI)	Upper LOA (95% CI)
Aortic Root (mm)	29.4 (9.4)	29.6 (8.8)	0.09 (−0.58; 0.76)	−2.87 (−4.03; −1.71)	3.05 (1.89; 4.21)
Ascending Aorta (mm)	27.0 (8.9)	26.0 (9.8)	−0.22 (−1.02; 0.59)	−3.86 (−5.25; − 2.47)	3.42 (2.03; 4.82)
Main Pulmonary Artery/Conduit (mm)	22.6 (6.5)	22.0 (6.6)	−0.20 (−1.18; 0.78)	− 4.30 (−6.00; − 2.09)	3.9 (2.20; 5.60)
Left Pulmonary Artery (mm)	15.6 (4.4)	16.2 (5.1)	0.95 (− 0.18; 2.09)	−4.05 (− 6.02; − 2.09)	5.96 (3.99; 7.93)
Right Pulmonary Artery (mm)	15.3 (4.0)	15.3 (4.2)	− 0.09 (− 0.93; 0.76)	− 3.91 (− 5.38; − 2.45)	3.74 (2.27; 5.20)
Descending Aorta (isthmus) (mm)	16.7 (4.8)	16.5 (4.8)	−0.32 (− 0.92; 0.28)	−2.98 (− 4.03; − 1.94)	2.35 (1.30; 3.39)
Left Anterior Descending Coronary Artery (mm)	3.8 (0.9)	3.8 (0.8)	0.03 (− 0.19; 0.24)	− 0.84 (− 1.21; − 0.47)	0.90 (0.52; 1.27)
Superior Vena Cava (mm)	16.4 (4.1)	15.9 (4.7)	−0.48 (− 1.00; 0.04)	−2.83 (− 3.73; − 1.93)	1.88 (0.98; 2.78)

*Abbreviations*: *mDixon* modified-Dixon; *bSSFP* balanced steady state free precision; *SD* standard deviation, *CI* confidence interval, *LOA* limit of agreement

Bias reflects mean difference for bSSFP - mDixon. LOA calculated as 1.96 x SD

### Time efficiency

The respective acquisition duration for the free-breathing, 135-slice whole heart sequences were comparable: 6.6 (2.6) minutes for the bSSFP and 7.4 (3.4) minutes for the mDixon, *(p = 0.20)*. The average navigator efficiency ranged between 40 and 60%.

## Discussion

This is the first study validating the mDixon whole-heart CMRA technique in CHD and aortic pathology. High quality mDixon CMRA images were obtained without the need for cardiac anesthesia, indicating that mDixon could be integrated into the CMR workflow, particularly for patients with expected flow and susceptibility artifact, systemic or pulmonary venous pathology.

Even though our pilot investigation warrants full-scale evaluation, our diverse patient sample supports the applicability of mDixon CMRA in patients of variable age, size and heart rate independent of prior catheter or surgical interventions. A wide spectrum of CHD is represented, including complex heterotaxy, different stages of single ventricle palliation, pulmonary venous abnormalities, valvular and great artery disease. All important anatomy was demonstrated with accurate morphologic detail. The datasets revealed consistent findings with regards to key reporting elements and they were deemed reliable for long-term surveillance and interpretation of changes over time for pre-procedural planning [3, 4].

In addition to diagnostic adequacy, we assessed a comprehensive range of image quality features and examined the accuracy of quantitative measurements in the coronary and peripheral chest vasculature between mDixon versus the two reference-conventioanl CMRA techniques, namely CE-CMRA and 3D whole-heart balanced bSSFP. In terms of visual quality scores, mDixon displayed adequate tissue characterization, sharper distal vascular anatomic borders and clearer spatial relationships among the extracardiac surrounding chest structures that interfere with fat tissue and air space within the field of view. Overall, the generated set of water images were characterized by robust homogeneous fat suppression, acceptable SNR, CNR [16, 29–31] and significantly less artifact burden. While conventional whole-heart techniques depend primarily on short T1-relaxation time and frequency-selective fat suppression, modified Dixon is a chemical shift-encoded fat-water separation methodology [17, 19]. Consequently, it is more resilient to background B0 and B1 magnetic field inhomogeneities that can be particularly strong in the thoracic cavity [20]. Due to ECG and respiratory gating, both whole heart sequences had minimal ghosting artifacts secondary to motion and spatial misregistration, [16, 32], though, mDixon demonstratesd significantly less sensitivity to blood flow and chemical shift distortions [21, 34]. The SNR has a complex dependency on the pulse sequence, parallel imaging factor, timing after contrast, and k-space parameters. The inherent SNR is higher for the bSSFP sequence, but the image noise is also higher. The expectation was that the bSSFP SNR would be higher, but the noise dominated and the SNR for the mDixon was higher.

Therefore, mDixon performance was superior in anatomic regions with disturbed blood flow, including stenotic and regurgitant valves, vascular anastomoses, stenotic or dilated vascular segments. Likewise, mDixon showed less susceptibility artifact induced by ferromagnetic implants as compared to bSSFP and CE-CMRA techniques. We observed smaller signal voids and superior fat suppression in the periprosthetic area in a variety of metal prostheses, from simple sternal wires and vascular plugs to artificial intracardiac valves, vascular stents and MRI compatible pacemakers. Experimental and limited adult publications on breast [34] and skeletal MRI image quality in the presence of metal prosthesis [26] have shown discrepant results between short tau inversion recovery (STIR) pulse and Dixon methodologies. Our study is the first to offer critical information on whole-heart sequence performance in the chest that is known for being technically challenging and prone to local magnetic field distortion. It is worth mentioning, that “any type of artifact” prevented us from performing a vascular measurement in 6 mDixon versus 4 bSSFP and 6 CE-CMRA images. Thus, metal interference occurred in only 2 out of the 6 mDixon cases as detailed previously.

Visualization of the vessel course and lumen patency are critically important in the CHD population for diagnosis and planning of transcatheter or surgical interventions. Such anatomic structures are challenging to delineate by conventional transthoracic echocardiography and invasive X-ray angiography has historically been the reference standard [35]. Geva et al. demonstrated adequate diagnostic capability of gadolinium-enhanced 3D CMRA in a large cohort of patients with congenital and acquired pulmonary and systemic venous abnormalities who had undergone cardiac catheterization [8]. In our study, the diagnostic yield of mDixon in depicting the pulmonary and neck veins was consistently comparable to that of the CE-CMRA and higher than the bSSFP. Our results are in line with existing adult literature showing high spatial resolution and vessel-to-background contrast in imaging of peripheral arteries via Dixon-based fat-free CMRA [24, 25]. The clinical relevance of applying mDixon sequences consists in providing high quality intracardiac information simultaneously with arterial and venous vascular data, including not only the pulmonary and neck veins, but also collateral vessels that play a pivotal role for targeted therapeutic interventions.

With regards to the coronary artery anatomy, both whole-heart methodologies allowed reliable identification of the origins and proximal courses with comparable fat suppression, contrast and vessel wall sharpness and overall visual quality scores. The visualization of the mid- to distal LAD was inadequate in 3 mDixon images versus none in the bSSFP. Heart rate did not seem to have any effect as the patients belonged to diverse age groups (4mo, 13 yrs., 27 yrs., 40 yrs). Three of those sequences were performed prior to the bSSFP. Additional analysis to further investigate the temporal effect of contrast showed no meaningful difference when mDixon was performed before the bSSFP. In the presence of contrast, both fat and blood demonstrate a significantly bright signal so fat suppression is important in delineating differences between tissues [36]. Currently, the contrast enhanced whole-heart bSSFP is the protocol of choice in pediatric and adult CHD coronary CMRA [3–5, 10, 14, 15, 37]. In a small pilot study of 8 healthy volunteers, Nezafat et al. demonstrated significantly higher CNR and SNR in the coronary artery tree with Dixon water-fat separation than spectral presaturation with inversion recovery (SPIR) fat suppression at 3 T, even without IV contrast administration [33]. mDixon techniques have been proven useful in peripheral CMRA as well [38, 39]. In contrast, Börnert et al., found that SNR and CNR tended to drop in bSSFP sequences after gadolinium administration and suggested that fat-saturated images may be advantageous in demonstrating small epicardial vessel structures and tissue boundaries [20]. Within the same context, we observed that the myocardium-blood pool contrast was significantly better in the bSSFP images due to smoother contrast intensity and somewhat finer delineation of the cardiac tissues. Given the advantageous perivascular fat suppression in combination with the possibility of contrast interference with visual quality, non-contrast mDixon CMRA could be a future consideration.

### Limitations

Our findings are limited by the retrospective study design and the relatively small sample size. Consequently, we refrained from performing certain quantitative statistical comparisons which would have required a larger cohort. The interrater agreement varied profoundly depending on the type of the sequence. It is plausible that the unblinded process could have generated observer bias. Interestingly, the kappa statistic was highest for the mDixon and lowest for the CE-CMRA and therefore, some of the discrepancy may be attributed to the different levels of experience between the reviewers when rating the best possible CE-CMRA quality images. mDixon works very well in our lab, however, generalizability of our results cannot be concluded. We attempted to maintain scanner settings similar for the two sequences to achieve comparable conditions; further manipulations may have had optimized some quality features. Despite the fact that our whole-heart scanning times are similar, we acknowledge that the mDixon images were acquired with a slightly higher parallel imaging factor that may degrade the image quality. This was done to make the image acquisition times approximately equal between the two sequences. Lastly, there is supporting evidence that cardiac fat carries important diagnostic information in differentiating pericardial or endo-myocardial fat infiltrative conditions [16]. Analysis of the mDixon fat-images was beyond the scope of this preliminary study, however, future studies should further investigate such source of information in the CHD population.

## Conclusion

In our pilot retrospective study, mDixon 3D ECG gated CMRA yielded precise delineation of complex cardiovascular anatomies in freely breathing patients with decreased artifacts compared to our standard bSSFP 3D ECG gated sequence. The mDixon technique performed comparably for evaluation of intracardiac and extracardiac anatomy. Importantly, mDixon suffered less artifact burden and may be an excellent alternative to bSSFP as the preferred strategy for gated angiography of CHD and aortopathy patients in the presence of disturbed blood flow, metallic implants, systemic or pulmonary venous disease.

## Additional files

Additional file 1: **Figure S1.** Example images from mDixon acquisition. The water, fat, in-phase, and opposed-phase images are shown in the panels A-D. Note the improved vessel delineation in the water image (A) secondary to fat signal suppression. (DOCX 230 kb)

Additional file 2: **Figure S2.** Average quality score for mDixon water and in-phase images. ***p-value <0.01. (DOCX 139 kb)

Additional file 3: **Figure S3.** Signal to noise and contrast to noise ratios for water and in-phase images. There is a significant difference in both parameters. ***p-value <0.001. (DOCX 187 kb)

Additional file 4: **Table S1.** Summary of agreement estimates for Image Quality measures: Average Quality Score, SNR and CNR. (DOCX 13 kb)

Additional file 5: **Table S2.** Agreement estimates for qualitative image quality measures. (DOCX 14 kb)

Additional file 6: **Table S3.** Quantitative comparison of the mDixon water and in-phase images for vessel measurements. (DOCX 16 kb)

## Data Availability

The dataset generated and/or analyzed during the current study are available from the corresponding author on reasonable request.

## Abbreviations

3D: Three dimensional
ASD: Atrial Septal Defect
AVSD: Atrio-Ventricular Septal Defect
AVV: Atrioventricular Valve
B/L: Bilateral
BAV: Bicuspid Aortic Valve
bSSFP: balanced Steady-State Free Precession;
CE: Contrast Enhanced
CHD: Congenital Heart Disease
CI: Confidence Interval
CMR: Cardiovascular Magnetic Resonance
CMRA: Cardiovascular Magnetic Resonance Angiography
CNR: Contrast-to-Noise Ratio
DORV: Double Outlet Right Ventricle
D-TGA: D-Transposition of the Great Arteries
ECG: Electrocardiogram
LAD: Left Anterior Descending coronary artery
LOA: Limit of Agreement
LPA: Left Pulmonary Artery
mDixon: modified Dixon
MPA: Main Pulmonary Artery
MV: Mitral Valve
PAPVC: Partial Anomalous Pulmonary Venous Connection
RPA: Right Pulmonary Artery
RV-PA: Right Ventricle to Pulmonary Artery;
S/P: Status Post
SCA: Subclavian Artery
SCMR: Society for Cardiovascular Magnetic Resonance
SD: Standard Deviation
SNR: Signal-to-Noise Ratio
SPIR: Spectral Presaturation with Inversion Recovery
STIR: Short tau Inversion Recovery
SVC: Superior Vena Cava
TAA: Transverse Aortic Arch
TAPVC: Total Anomalous Pulmonary Venous Connection
TOF: Tetralogy of Fallot
VSD: Ventricular Septal Defect
