# Supplementary material for: mDixon ECG-gated 3-dimensional cardiovascular magnetic resonance angiography in patients with congenital cardiovascular disease
Source: J Cardiovasc Magn Reson. 2019 Aug 8;21:52. doi: 10.1186/s12968-019-0554-3 (PMC6686451; doi:10.1186/s12968-019-0554-3)
Supplement: Supplementary file 1 — Figure S1. Example images from mDixon acquisition. The water, fat, in-phase, and opposed-phase images are shown in the panels A-D. Note the improved vessel delineation in the water image (A) secondary to fat signal suppression. (DOCX 230 kb) [file 12968_2019_554_MOESM1_ESM.docx]

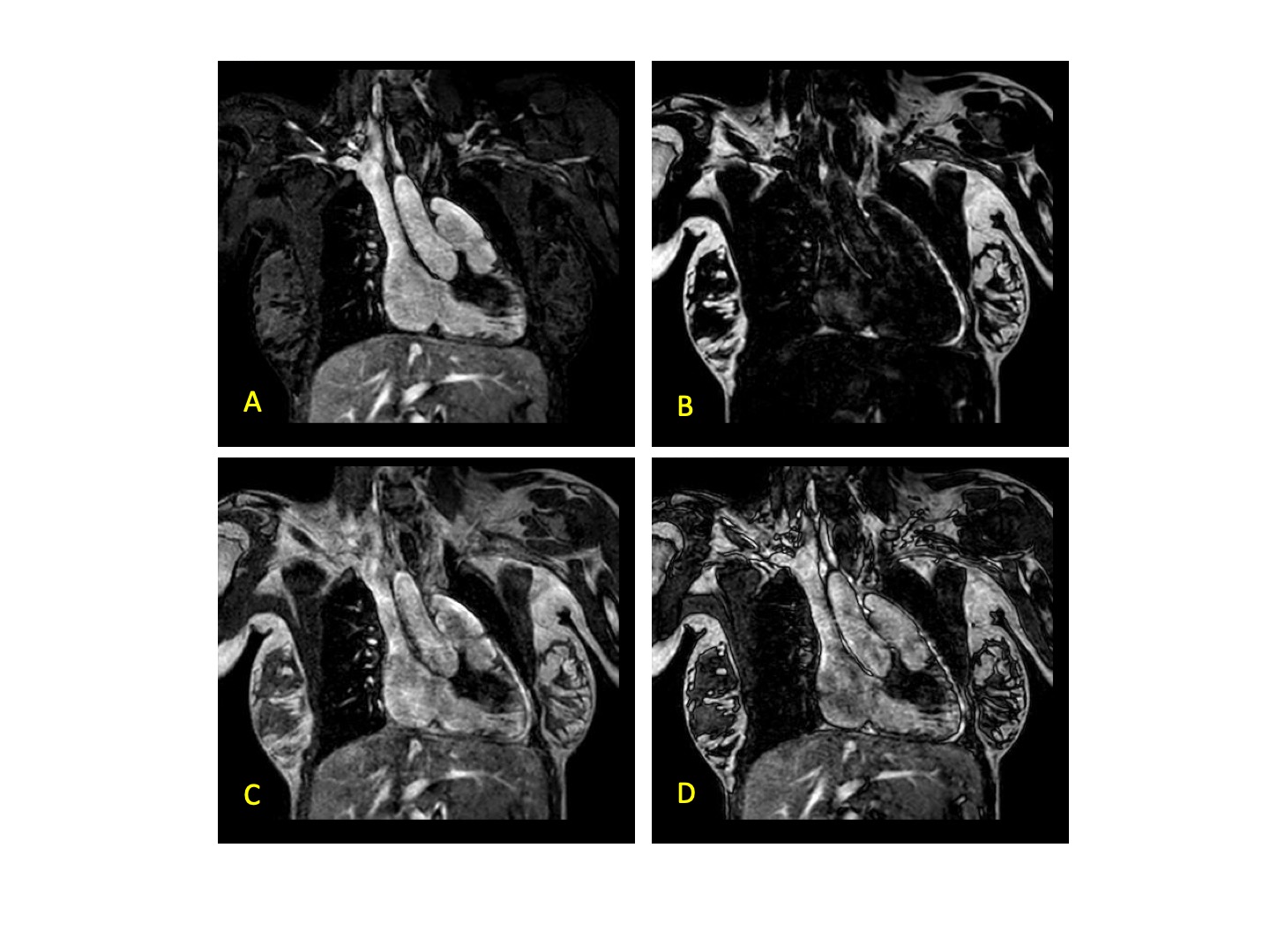


Supplemental Figure S1. Example images from mDixon acquisition. The water, fat, in-phase, and opposed-phase images are shown in the panels A-D. Note the improved vessel delineation in the water image (A) secondary to fat signal suppression.
