# Supplementary figures and images for: mDixon ECG-gated 3-dimensional cardiovascular magnetic resonance angiography in patients with congenital cardiovascular disease
Source: J Cardiovasc Magn Reson. 2019 Aug 8;21:52. doi: 10.1186/s12968-019-0554-3 (PMC6686451; doi:10.1186/s12968-019-0554-3)

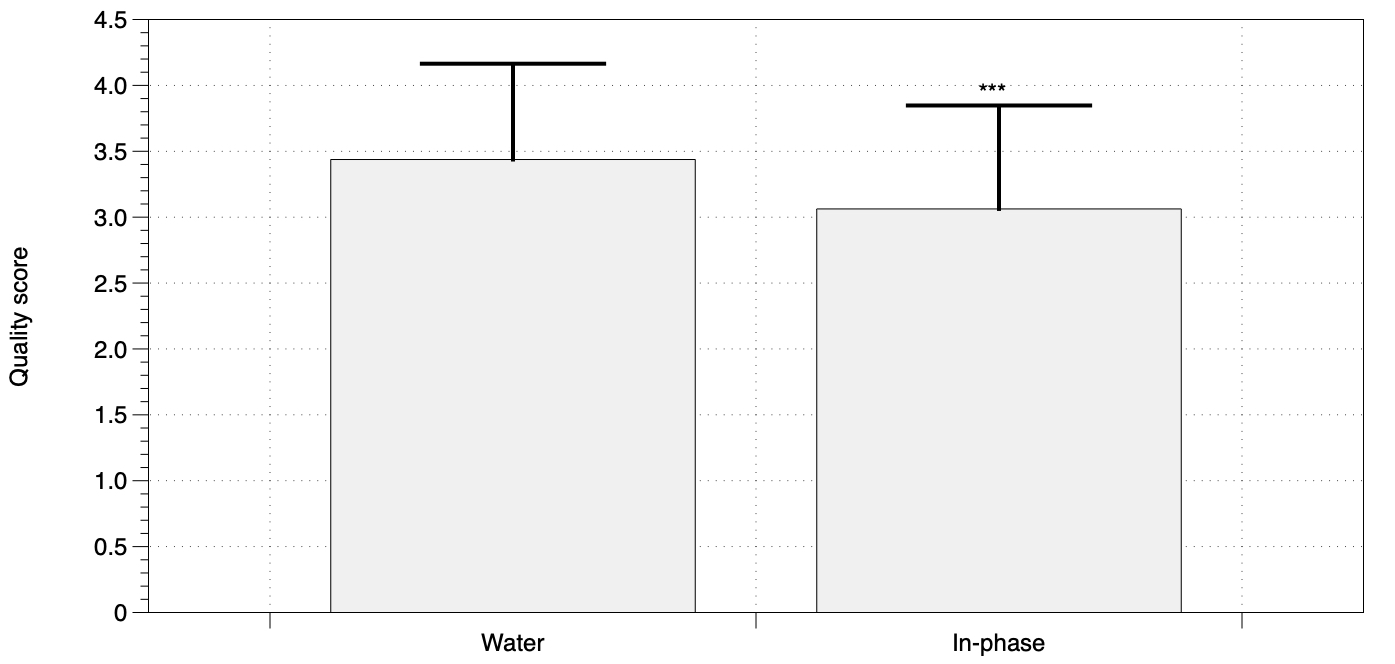


Supplemental Figure S2. Average quality score for mDixon water and in-phase images. ***p-value <0.01.

Supplement: Supplementary file 2 — Figure S2. Average quality score for mDixon water and in-phase images. ***p-value <0.01. (DOCX 139 kb) [file 12968_2019_554_MOESM2_ESM.docx]
