# Supplementary material for: mDixon ECG-gated 3-dimensional cardiovascular magnetic resonance angiography in patients with congenital cardiovascular disease
Source: J Cardiovasc Magn Reson. 2019 Aug 8;21:52. doi: 10.1186/s12968-019-0554-3 (PMC6686451; doi:10.1186/s12968-019-0554-3)
Supplement: Supplementary file 3 — Figure S3. Signal to noise and contrast to noise ratios for water and in-phase images. There is a significant difference in both parameters. ***p-value <0.001. (DOCX 187 kb) [file 12968_2019_554_MOESM3_ESM.docx]

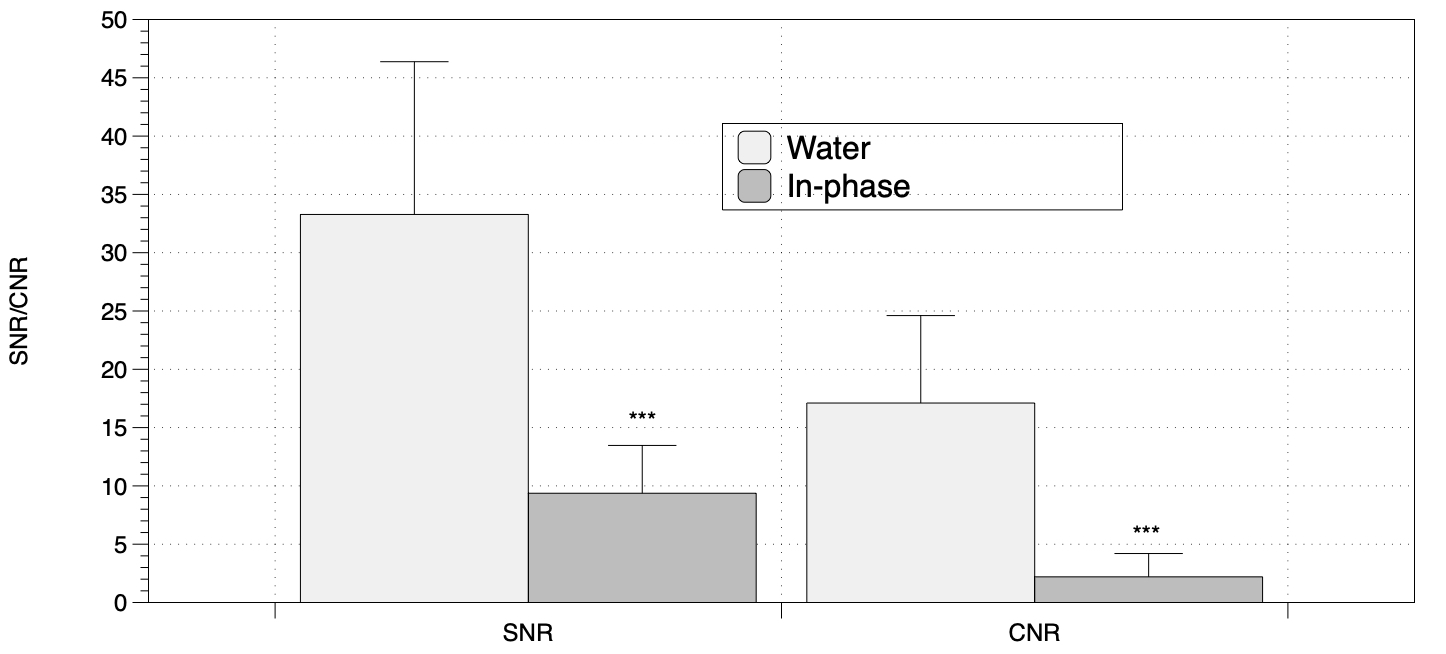


Supplemental Figure S3. Signal to noise and contrast to noise ratios for water and in-phase images. There is a significant difference in both parameters. ***p-value <0.001.
