# Supplementary material for: mDixon ECG-gated 3-dimensional cardiovascular magnetic resonance angiography in patients with congenital cardiovascular disease
Source: J Cardiovasc Magn Reson. 2019 Aug 8;21:52. doi: 10.1186/s12968-019-0554-3 (PMC6686451; doi:10.1186/s12968-019-0554-3)
Supplement: Supplementary file 4 — Table S1. Summary of agreement estimates for Image Quality measures: Average Quality Score, SNR and CNR. (DOCX 13 kb) [file 12968_2019_554_MOESM4_ESM.docx]

Table S1. Summary of agreement estimates for Image Quality measures: Average Quality Score, SNR and CNR.

| **Image Quality** | **mDixon**  Mean (SD) | **bSSFP**  Mean (SD) | **Bias**  (95% CI) | **Lower LOA**  (95% CI) | **Upper LOA**  (95% CI) |
| --- | --- | --- | --- | --- | --- |
| **Average Quality Score** | 3.40 (0.70) | 3.20 (0.50) | -0.21 (-0.50; 0.08) | -1.56 (-2.07; -1.06) | 1.14 (0.64; 1.65) |
| **SNR** | 33.30 (13.10) | 30.60 (12.20) | -2.68 (-7.57; 2.22) | -25.41 (-33.89; -16.92) | 20.05 (11.56; 28.54) |
| **CNR** | 17.10 (7.50) | 19.70 (7.70) | 2.58 (-0.64; 5.80) | -12.37 (-2.07; -1.06) | 1.14 (0.64; 1.65) |
| Abbreviations: mDixon: modified -Dixon; bSSFP, balanced steady state free precision; SD, standard deviation, CI, confidence interval; LOA, limit of agreement  Notes: Bias reflects mean difference for balanced SSFP - mDixon. LOA calculated as 1.96 x SD. | | | | | |
