# Supplementary material for: mDixon ECG-gated 3-dimensional cardiovascular magnetic resonance angiography in patients with congenital cardiovascular disease
Source: J Cardiovasc Magn Reson. 2019 Aug 8;21:52. doi: 10.1186/s12968-019-0554-3 (PMC6686451; doi:10.1186/s12968-019-0554-3)
Supplement: Supplementary file 5 — Table S2. Agreement estimates for qualitative image quality measures. (DOCX 14 kb) [file 12968_2019_554_MOESM5_ESM.docx]

Table S2. Agreement estimates for qualitative image quality measures.

| **Image Quality** | **mDixon**  N (%) | **bSSFP**  N (%) | **CE-MRA**  N (%) | **Agreement**  **mDixon│SSFP** | **Agreement**  **mDixon│MRA** | **Agreement**  **bSSFP│MRA** |
| --- | --- | --- | --- | --- | --- | --- |
| **Ability to Visualize Pulmonary Veins** | 22 (91.7%) | 18 (75%) | 23 (95.8%) | 20 (83.3%) | 21 (87.5%) | 21 (87.5%) |
| **Ability to Visualize Neck Veins** | 22 (91.7%) | 20 (83.3%) | 23 (95.8%) | 18 (75%) | 21 (87.5%) | 19 (79.2%) |
| **Artifact Preventing Vascular Measurement** | 6 (3%) | 4 (2%) | 6 (3%) | 186 (97.9%) | 184 (96.8%) | 188 (97.9%) |
| Abbreviations: mDixon: modified-Dixon; bSSFP: balanced Steady State Free Precision; CE-MRA: Contrast-Enhanced MRA  N represents the number of cases out of the total number of cases (N=24) that pulmonary veins or neck veins were visualized, or the number of vascular measurements not performed due to artifact out of the total number of measurements (N=192). | | | | | | |
