# Supplementary material for: mDixon ECG-gated 3-dimensional cardiovascular magnetic resonance angiography in patients with congenital cardiovascular disease
Source: J Cardiovasc Magn Reson. 2019 Aug 8;21:52. doi: 10.1186/s12968-019-0554-3 (PMC6686451; doi:10.1186/s12968-019-0554-3)
Supplement: Supplementary file 6 — Table S3. Quantitative comparison of the mDixon water and in-phase images for vessel measurements. (DOCX 16 kb) [file 12968_2019_554_MOESM6_ESM.docx]

| **Cross-sectional Vessel Diameter** | **mDixon - water**  Mean (SD) | **mDixon – in phase**  Mean (SD) | **Bias**  (95% CI) | **Lower LOA**  (95% CI) | **Upper LOA**  (95% CI) |
| --- | --- | --- | --- | --- | --- |
| **Aortic Root (mm)** | 29.4 (9.4) | 29.6 (9.7) | -0.45  (-0.56; -0.34) | -3.34  (-4.03; -2.57) | 2.43  (1.66; 3.20) |
| **Ascending Aorta (mm)** | 27.0 (8.9) | 25.8 (10.3) | 0.38  (0.23; 0.52) | -3.58  (-4.6; -2.56) | 4.33  (3.31; 5.35) |
| **Main Pulmonary Artery/Conduit (mm)** | 22.6 (6.5) | 23.1 (7.3) | -0.2  (-0.31; -0.08) | -3.26  (-3.96; -2.35) | 2.75  (1.94; 3.56) |
| **Left Pulmonary Artery (mm)** | 15.6 (4.4) | 15.5 (4.2) | 0.26  (0.18; 0.34) | -1.80  (-2.34; -1.27) | 2.33  (1.79; 2.87) |
| **Right Pulmonary Artery (mm)** | 15.3 (4.0) | 15.5 (4.5) | -0.045  (-0.13; 0.04) | -2.27  (-2.85; -1.69) | 2.18  (1.60; 2.86) |
| **Descending Aorta (isthmus) (mm)** | 16.7 (4.8) | 16.8 (4.9) | -0.26  (-0.35; -0.17) | -2.71  (-3.35; -2.07) | 2.19  (1.55; 2.83) |
| **Left Anterior Descending Coronary Artery (mm)** | 3.8 (0.9) | 3.9 (0.9) | -0.009  (-0.035; 0.02) | -0.66  (-0.85; -0.48) | 0.64  (0.46; 0.83) |
| **Superior Vena Cava (mm)** | 16.4 (4.1) | 16.6 (4.4) | 0.62  (-0.40; 0..85) | -6.63  (-7.06; -3.91) | 6.74  (5.16; 8.31) |
| Abbreviations: mDixon: modified Dixon; bSSFP**:** balanced steady state free precision; SD**:** standard deviation, CI**:** confidence interval; LOA**:** limit of agreement. Bias reflects mean difference for bSSFP - mDixon. LOA calculated as 1.96 x SD. | | | | | |

Supplemental Table S3. Quantitative comparison of the mDixon water and in-phase images for vessel measurements.
